# Spatial distribution and risk assessments due to the microplastics pollution in sediments of Karnaphuli River Estuary, Bangladesh

**DOI:** 10.1038/s41598-022-12296-0

**Published:** 2022-05-20

**Authors:** Md. Refat Jahan Rakib, M. Belal Hossain, Rakesh Kumar, Md. Akram Ullah, Sultan Al Nahian, Nazmun Naher Rima, Tasrina Rabia Choudhury, Samia Islam Liba, Jimmy Yu, Mayeen Uddin Khandaker, Abdelmoneim Sulieman, Mohamed Mahmoud Sayed

**Affiliations:** 1 Department of Fisheries and Marine Science, Faculty of Science, Noakhali Science and Technology University, Noakhali, Bangladesh; 2 School of Engineering and Built Environment, Griffith University, Brisbane, QLD Australia; 3 School of Ecology and Environment Studies, Nalanda University, Rajgir, Bihar 803116 India; 4 Bangladesh Oceanographic Research Institute, Cox’s Bazar, Bangladesh; 5 Analytical Chemistry Laboratory, Chemistry Division Atomic Energy Centre Dhaka, Bangladesh Atomic Energy Commission, Dhaka, 1000 Bangladesh; 6 Materials Science Division, Atomic Energy Centre Dhaka, Bangladesh Atomic Energy Commission, Dhaka, 1000 Bangladesh; 7 Centre for Applied Physics and Radiation Technologies, School of Engineering and Technology, Sunway University, 47500 Bandar Sunway, Selangor Malaysia; 8 Department of Radiology and Medical Imaging, Prince Sattam Bin Abdulaziz University, 11942 Alkharj, Saudi Arabia; 9 Faculty of Engineering and Technology, Future University in Egypt, New Cairo, 11835 Egypt; 10 Environmental and Life Sciences, Faculty of Science, Universiti Brunei Darussalam, Gadong, Brunei Darussalam

**Keywords:** Environmental sciences, Environmental social sciences, Hydrology, Limnology, Natural hazards, Solid Earth sciences

## Abstract

Microplastics (MPs) have become an emerging global pollutant due to their widespread dispersion and potential threats to marine ecosystems. However, studies on MPs in estuarine and coastal ecosystems of Bangladesh are very limited. Here, we conducted the first study on abundance, distribution, characteristics, and risk assessment of microplastics in the sediment of Karnaphuli River estuary, Bangladesh. Microplastic particles were extracted from sediments of 30 stations along the estuary by density separation and then enumerated and characterized using a stereomicroscope and Fourier Transform Infrared (FT-IR) spectroscopy. In the collected sediment of the Karnaphuli River estuary, the number of MPs varied from 22.29 to 59.5 items kg^−1^ of dry weight. The mean abundance was higher in the downstream and left banks of the estuary, whereas the predominant shape, colour, and size of MPs were films (35%), and white (19%), and 1–5 mm (30.38%), respectively. Major polymer types were polyethylene terephthalate, polystyrene, polyethylene, cellulose, and nylon. MPs were found to pose risks (low to high) in the sediment of the estuary, with the highest risk occurring at one station near a sewage outlet, according to the results of risk analyses using the pollution risk index, polymer risk index (H), contamination factors, and pollution load index (PLI). The single value index, PLI, clearly demonstrated that all sampling sites were considerably polluted with microplastics (PLI > 1). H values showed toxic polymers, even in lower proportions, possess higher polymeric hazard scores and vice versa. This investigation uncovered new insights on the status of MPs in the sediments of the Karnaphuli River estuary, laying the groundwork for future research and control of microplastic pollution and management.

## Introduction

Marine and coastal ecosystems are constantly victims of continuous and accumulative stress from human and industrial activities. As a result of human and industrial activities, about 5–12 million tonnes of plastic waste enters various marine habitats each year, both from land and marine-based sources^1–3^. Plastics account for a significant portion of marine debris, and it has become an ever-increasing marine environmental concern owing to ecological and biological hazards^4^. This geological marker is ubiquitous and found in various sizes, from meters to micrometers^5,6^. Plastics are very resistant to biodegradation and break down into smaller pieces due to weathering, UV radiation, mechanical abrasion, and photo-degradation^7–9^.

National Oceanic and Atmospheric Administration (NOAA) referred to plastic particles less than 5 mm as microplastics (MPs). These microplastics include high-density polyethylene (PE), low-density polyethylene (LDPE), polypropylene (PP), polystyrene (PS), polyvinylchloride (PVC), polyethylene terephthalate (PET), and Polyurethane (PUR) resins; and polyester, polyamide, and acrylic (PP&A) fibers^9^. MPs in the environment are sourced from cosmetics, paints, scrubbing agents, and industrial effluents (primary microplastics) or by-products of larger size plastics degradation/breakdown^10^. Due to their tiny sizes, these hazardous elements are deliberated bioavailable to organisms over the food web. Ingestion of microplastics may thus be responsible for bringing toxins to the base of the food chain, where bioaccumulation may occur^11^.

MPs may persist for hundreds to thousands of years, and their accumulation poses a severe hazard to aquatic and terrestrial ecosystems, biota, and human health^12,13^. MPs are consumed and uptake as food by bivalves, zooplankton, mussels, fishes, shrimps, and other marine vertebrates and invertebrates, whether filter- or deposit-feeding^14–16^. These incidents further lead to physical damage and health hazards, such as gastrointestinal cuts or obstructing intestinal systems, reduced growth rate, reproductive problems, blocked enzyme production, pathological and oxidative stress^10,17,18^. As a result, MPs may readily transfer to the human body via seafood has raised concern worldwide.

Over the last five years, studies on MPs pollution have increased dramatically worldwide, realizing their harmful effects on ecosystems and organisms. Numerous investigations have already proved that extent of MPs pollution in estuarine waters is very high in various parts of the world, such as Yellow River estuary, China^19^, Shanghai estuary, China^20^, Bohai Bay, China^21^ and coastline along the Persian Gulf, Iran^22^. Bangladesh is one of the major plastic-producing countries globally, with over three thousand small and big plastic industry sites^23^. The per capita consumption of plastics in Bangladesh has drastically increased to 3.5 kg, with a total production of 3000 tons of plastic debris each day^23^. In addition, the percentage of contribution to mismanaged plastic waste in global total is very high in Bangladesh^3,23^ because a large part of this waste is released to the environment without any kind of recycling. Despite this, only a few studies have been conducted on the status and risk assessment of MPs pollution in Bangladesh. High abundance of MPs was determined in the sediments of Cox’s Bazar^1,2,23^, in salt pans from Moheskhali channel^24^, in tiger shrimp, brown shrimp, Bombay-duck, and gold-stripe sardine from the northern Bay of Bengal^25,26^. As rivers and estuaries are the main sources of land-based MPs flowing into the ocean, investigating the abundance, distribution, and potential risks of MPs in estuarine, marine, and coastal ecosystems is necessary for adequately maintaining the estuary ecosystems and human health.

Karnaphully River estuary is one of the significant urban estuaries in the Chittagong seaport area beside the Bay of Bengal. Karnaphully river estuary is a lifeline of Bangladesh’s economic wheel; a major seaport is highly dependent on the river. Every month more or less 4000 ships and 10,000 registered and unregistered vessels ply the river. Apart from them, shipbreaking industry, oil refineries, and fertilizer industry, more than 1000 small and heavy industries are standing on both sides of the river. More than 2500 tonnes of garbage from nearly 6 million city dwellers flows into the Karnaphuli River daily through different canals and drains due to poor sewer systems. Recently, Chittagong Port Authority found a layer of 2–7 m polythene during dredging. It is documented that, Karnaphuly is the worst victim of uncontrolled pollution and lack of proper waste management, posing a severe environmental threat. Earlier environmental studies of the Karnaphuli estuary have mainly focused on water quality^27^, microbial loads^28^, heavy metals^29^, and radioactivity^30^, but the MPs pollution has also not received any attention. Unfortunately, there is no information concerning the microplastic pollution status in the sediment of the Karnaphuli estuary. Therefore, the aims of this study are (i) to determine the MPs loading and their distributional pattern along the Karnaphuli estuary, (ii) to identify shapes, size, and polymeric characteristics of MPs; and (iii) to assess the potential risks of MPs through multiple indices. This research appears to be the first comprehensive investigation on the Karnaphuli River estuary, which assessed the ecological risks posed due to the MPs pollution.

## Materials and methods

### Study sites

Karnaphuli is the second-largest estuary in Bangladesh. The Karnaphuli river flows from the Lushai hills in Mizoram, India, and has a watershed area of nearly 11,000 km^2^ ^2,31^; and travels 180 km of Rangamati mountainous wilderness, Bangladesh. Further, the Karnaphuli River estuary flows about 170 km via the port city of Chittagong and ends in the Bay of Bengal^32^. The estuary is abundant with semidiurnal tides of 2–4 m range with a mean depth of 8–10 m in the external zone of the river^33^. The environmental behavior of the Karnaphuli River estuary changes periodically due to the Indian monsoon^31^. Because of the seasonal wind movement, the climatic conditions of the riverine area in Chittagong vary from season to season. The monsoon season is hot, gloomy, and oppressive, whereas the dry season is warm, generally clear, and humid. The temperature ranges from 58 °F to 90 °F, with an annual rainfall of 17.8 inches^32^. The entire river watershed is geologically composed of tertiary rocks covered with alluvial deposits, with successive layers of mud and sand^34^.

### Sample collection and processing

**Figure 1:**
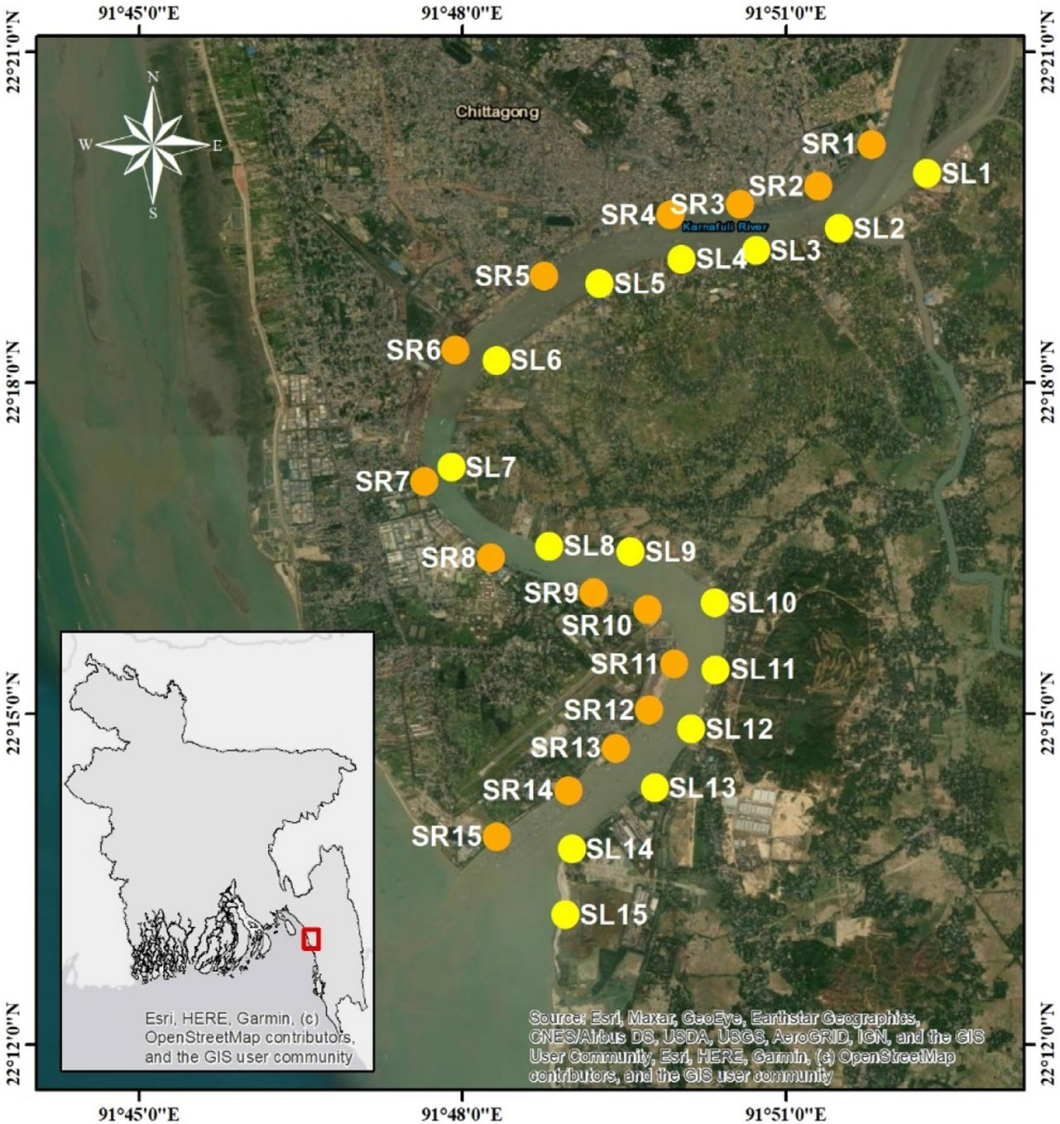
The study area and sampling design in the Karnaphuli River estuary. This map was constructed using ArcGIS 10.7.

Sediment samples were collected across upstream, midstream, and downstream of the river. From July 2020 to May 2021, 90 sediment samples were collected using an Ekman dredge at low tide from 30 locations on the left and right banks of the Karnaphuli River estuary in Bangladesh (Fig. 1). All sediment samples were collected in replicates. Sediment samples, nearly 1 kg weight, were processed at the Bangladesh Oceanographic Research Institute laboratory. Each sediment sample was frozen and dried before sieving with 5 mm to remove larger plastic particles^35^.

### Sample preparation and extraction

MPs were extracted from 90 sieved sediment samples (45 sediment samples from each bank) according to the NOAA procedure for bed sample analysis^36^. Sediment samples were first homogenized with a stainless-steel spoon before being dried at 90 °C for 24 h. Dried samples were mixed with 300 mL of ZnCl_2_ (1.5 g /mL) salt solution^37^ and stirred continuously. After that, all floating solids were sieved using 0.3 mm sieve and kept in 20 mL each of 30% H_2_O_2_ and FeSO_4_ (0.05 M) solution for oxidation of organic materials^38–40^. The wet peroxide oxidation treatment was performed to remove organic contaminants at approximately 75 °C for 5 min and boiled till no organic material was left. Then, 6 g sodium chloride (NaCl) per 20 mL of sample solution using density separation method and heated at 75 °C to dissolve sodium chloride entirely and placed to settle using density separator for 24 h. Floating solids were collected using a 0.3 mm custom sieve, and the density separator was rinsed thoroughly with distilled water to transfer all. The solution from the density separator was filtered using 0.45 μm mesh size cellulose nitrite filter paper^41^.

### Microplastics identification and characterization

All extracted MPs from sediment samples were identified, counted, and photographed under dissecting light stereomicroscope (Olympus SZX16, Germany) with 10 × to 1000 × magnification, and further, particles were analyzed based on their colors, sizes, and shapes. Shapes of MPs were categorized as fragments, films, foams, fishing lines/fibers, pellets/granules, or flakes. MPs were classified into four size classes according to their lengths: > 5000 μm, 5000–1000 μm, 1000–250 μm, and 250–125 μm^42^. Polymeric analysis of MPs was performed using μ-Fourier Transform Infrared Spectrometer (μ-FTIR) (Perkin-Elmer instruments, STA), equipped with attenuated total reflection (ATR) has been done at room temperature (300 K), in the range of 400–1600 cm^−1^ wavenumber.

### Contamination factors, pollution load index, and polymeric and pollution risk assessments

MPs contamination factor (CF), pollution load index (PLI), pollution risk index (PRI), and polymeric risk assessment (H) in the river sediment were estimated as described in previous studies^24,43,44^. Tomlinson et al.^45^ proposed the contamination factors (CF) and pollution load index (PLI) to assess the pollution level in natural ecosystems. CF, PLI, PRI, and H were categorized as values from low contamination to very high contamination, described in Table 1^24,43,44^.

**Table 1:** Category engaged in the MPs contamination factors (MPCFs).

CF	Risk category	H	Risk category	PRI	Risk category	PLI	Risk category
< 1	Low contamination	< 10	I	< 150	Low	> 1	Polluted
1–3	Moderately contamination	10–100	II	150–300	Medium		
3–6	Considerably contamination	101–1000	III	300–600	Considerable		
≥ 6	Very highly contamination	1001–10,000	IV	600–1200	High		
		> 10,000	V	> 1200	Very high		

The PLI is regarded as a standardized protocol for assessment and monitoring the extent of pollution between different areas^45^. Sampling sites are considered to be polluted, as PLI > 1^45^. The risk assessment model was as follows^45^:

1 $${\text{CF}}_{{\text{i}}} = \frac{{{\text{C}}_{{\text{i}}} }}{{{\text{C}}_{{\text{o}}} }}$$

2 $${\text{PLI}}_{{{\text{river}}}} = \sqrt[{\text{n}}]{{{\text{CF}}_{1} \times {\text{CF}}_{2} \times {\text{CF}}_{3} \cdots \times {\text{CF}}_{{\text{n}}} }}{ }$$

where i denotes a station, n presents the number of stations, C_i_ represents MPs abundance at ith station, and C_o_ is the minimum baseline concentration in rivers. Though, the least MPs abundance found in this investigation was considered as minimum baseline concentration due to the lack of previous background data in the same environments and the analytical context of this study.

Xu et al.^46^ evaluated that MPs concentration and chemical composition need to be considered to evaluate the potential risks in surface sediments^46^. Lithner et al.^47^ assigned hazard scores to compute polymeric risks based on their toxicity level in respective ecosystems. Polymeric risks can be estimated using chemical toxicity coefficients or risk scores (S_j_) for the identified polymers in the sediment samples. The hazard scores for MP polymers identified were PS = 30; PE = 11; PET = 4; and Nylon = 50, while cellulose was not available and was excluded in the calculation. Equations (3) and (4) presented polymeric risk assessment for different sampling sites and the entire Karnaphuli River Estuary, Bangladesh, respectively. P_ji_ denotes the number of and S_j_ presents risk score for each single MPs polymer recognized at ith station^24,47^, whereas H_river_ is calculated as the nth root of the polymer risk indices products (Eq. 4), as follows:

3 $${\text{H}}_{{\text{i}}} = \sum {\left( {\frac{{{\text{p}}_{{{\text{ji}}}} }}{{{\text{C}}_{{\text{i}}} }}{ } \times {\text{S}}_{{\text{j}}} } \right)}$$

4 $${\text{H}}_{{{\text{river}}}} = ({\text{H}}_{{\text{A}}} \times {\text{ H}}_{{\text{B}}} \times {\text{H}}_{{\text{C}}} \times {\text{H}}_{{\text{D}}} \times \cdots {\text{H}}_{{\text{n}}} )^{{1/{\text{n}}}}$$

To compute MPs pollution risk index (PRI), Kabir et al.^44^ developed a pollution risk formula for particular stations as well as an entire river; Eqs. (5) and (6) are described as follows:

5 $${\text{PRI}}_{{\text{i}}} = {\text{H}}_{{\text{i}}} \times {\text{CF}}_{{\text{i}}}$$

6 $${\text{PRI}}_{{{\text{river}}}} = ({\text{PRI}}_{{1}} \times {\text{PRI}}_{{2}} \times {\text{PRI}}_{{3}} \times \cdots {\text{MPRI}}_{{\text{n}}} )^{{{1}/{\text{n}}}}$$

### Quality control

All equipment needs to be rinsed using de-ionized water before and after use. All stock solutions were filtered using 0.45 μm mesh size filter paper before use to avoid MPs contamination. Also, all the glassware was rinsed thrice with purified water. All samples were kept covered with aluminum foil or glassware whenever possible or under analysis to avoid external MPs contamination. Three blank samples without salt were analyzed simultaneously to correct any possible MPs contamination from sample processing. No MPs were found in blank samples. Polyester-type clothing was avoided to ensure intentional contamination of microplastics in the samples, and during handling, cotton-made laboratory aprons and nitrile gloves were used. All non/plastic sieves were washed and sonicated properly, before and after use. All MPs samples were kept in Petri dishes and appropriately covered with aluminum foil; then, all Petri dishes were placed in a glass desiccator to avoid airborne MPs contamination.

### Statistical analysis and image preparation

Data were expressed in mean values with their respective standard deviation for better interpretation of MPs pollution. Statistical analyses were performed using R (Studio v.1.1.453, PAST), whereas graphics elaboration was conducted on Prism 8.0 (Graph Pad Software Inc., USA), and ArcGIS 10.6 was used for MPs mapping. One-way analysis of variance (ANOVA) was performed, using Origin Pro, between two or more samples of both left and right banks for investigating their significance (significance level of 0.001). Principal component analysis (PCA) was also performed between independent indicators, in which raw data for MPs abundance on both right and left banks were analyzed to establish a relationship between MPs and sampling sites. Besides, a systematic cluster, i.e., the Heat map analysis method was performed for sampling sites to determine pollution characteristics and sources of pollution.

## Results and discussion

### Occurrence and abundance of MPs in sediments

**Figure 2:**
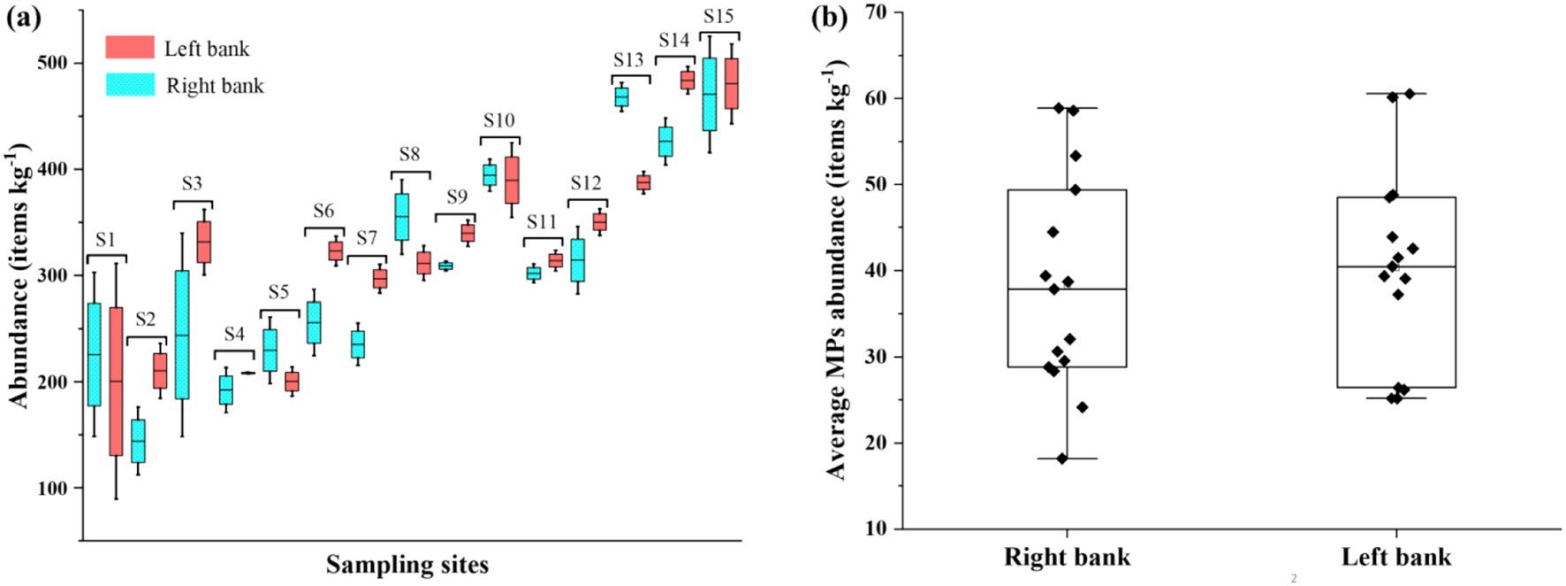
MPs abundance (**a**) at an individual bank and (**b**) overall average in sediments of Karnaphuli river estuary.

Total abundance of MPs from 30 sampling sites (15 stations from each bank) of the Karnaphuli River estuary was 1177 items kg^−1^ DW. MPs abundance varied in the range of 22.29–59.5 item kg^−1^ of dry weight (DW) of sediments in the Karnaphuli River estuary. The upstream presented lower MPs abundance (26.8, 22.3, 25.2, and 27 item kg^−1^ DW recorded from stations 1, 2, 4, and 5, respectively). Average MPs concentrations were 39.2 ± 11.6 and 40.32 ± 11.41 item kg^−1^ of dry weight at the right bank (SR) and left bank (SL) sampling sites, respectively (Fig. 2b). MPs abundance was observed maximum at site 15 (S15) of the left bank with 59.5 items·kg^−1^ DW, followed by 56.9 and 53.54 items kg^−1^ DW at sites 14 and 13 of the left bank, respectively. Figure 2a clearly presented increasing trends for MPs abundance from upstream to downstream in sediments. The lowest MP abundance was found at station S2 (22.3 particles kg^−1^ DW) (Fig. 2a). However, MPs abundance increased with the downstream and observed maximum at the S15 sampling site. All three sites were in the mouth of the river, which is connected to the sea directly. These results showed that MPs abundance in the sediment increased towards the mouth of the river estuary. This condition might emerge due to the estuary position, which is influenced because of fluctuating currents, tides, and direct access to the sea^48,49^. MPs particle distribution path and trajectory are influenced due to hydrodynamic force, i.e., flow velocity in the estuary as it enters the marine environment^50^. Therefore, the estuaries are prone to MPs contamination and should be considered hotspots for MPs sinks^51,52^.

The main reasons behind significant MPs pollution in Karnaphuli river estuary regions are high industrial effluents and municipal sewage generation rate^53,54^. However, there are no MPs pollution studies in the neighboring areas. Insufficient and inadequate infrastructure and sewage and industrial waste management services have caused a high abundance and disposition of MPs. Still, lack of community involvement has become an additional significant concern in reducing solid waste. For example, Chowdhury et al.^54^ reported that municipal solid waste increased from 538 tons/day in 1999 to 1890 tons/day in 2009 due to inadequate segregation of municipal solid waste in residential communities of Chittagong City. Also, the World Bank Group^55^ reported that municipal solid waste management service in Bangladesh is yet to be improved with suitable infrastructure and services. Therefore, Mediana and Gamse^56^ observed that significant quantities of solid waste, including plastic waste, be disposed of into rivers of Bangladesh. Furthermore, Karnaphuli River Estuary receives solid waste disposal sites from densely populated human settlements and industrial activities in Chittagong City^54^.

**Figure 3:**
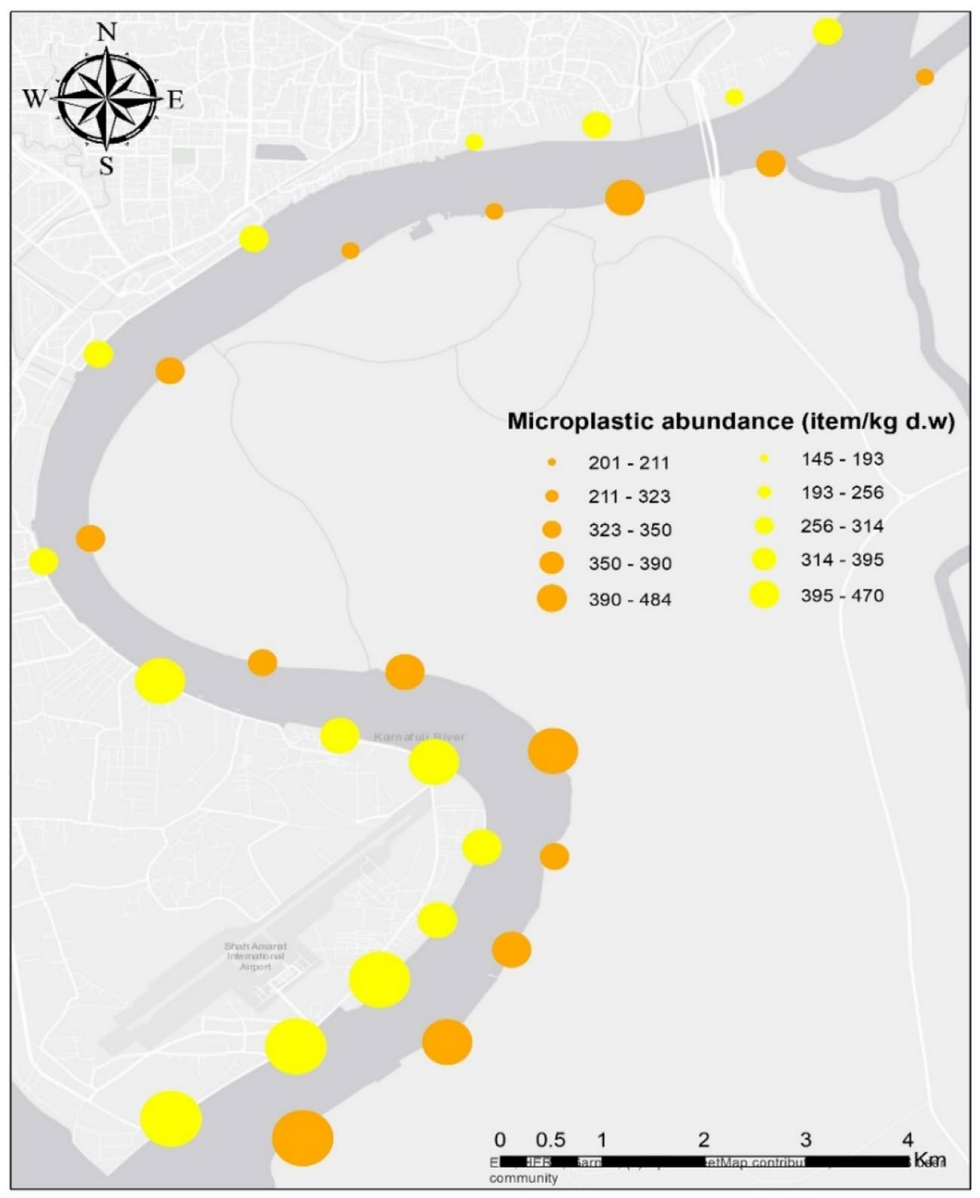
Spatial distribution of MPs among the sampling sites of Karnaphuli River estuary. This map was constructed using ArcGIS 10.7.

Existing riverine MPs research investigated spatial mapping of plastics across river channels. Microplastics enter the Karnaphuli River estuary from various point and non-point (or diffuse) sources. Point sources are from direct discharge, e.g., wastewater effluents, drainage ditches, agricultural runoff, and storm drains, whereas non-point sources are littering, spread over large areas^57^. The high abundance of MPs at the river downstream is significantly influenced by massive discharge by wastewater treatments and sewage effluents via agricultural, industrial, and urbanized areas due to population density and catchment size (Fig. 3). Moderate MPs pollution was observed frequently in sediments, from sampling sites of S1, S2, S4, and S5 due to household activities, microbeads, commercial fishing, etc. Assessing MPs from wastewater treatment plants, the equivalent population serviced and utilized via treatment methods, e.g., tricking filters, activated sludge, etc., needs to be considered^58^. Besides, huge amounts of MPs are consistently observed in close proximity to plastic manufacturing and industrial units. Andrady^8^ reported that MPs observed in terrestrial and aquatic ecosystems mainly belong from blast/raw materials used in industrial processes, either from unregistered discharge or accidental leakages. Several studies investigated the abundance of MPs in riverine sediments^59,60^. MPs variations between banks of river estuary were analyzed using one-way Analysis of Variance (ANOVA). Which was paired using Tukey’s HSD test. Results showed that MPs abundance were not significantly differed (F = 0.2465, df = 1, *p* < 0.01) among fifteen sampling sites (Table 2). Tukey’s test indicated that the difference of the means is not significant at the significance level of 0.01.

**Table 2:** MPs abundance analysis between banks of river using ANOVA and Tukey’s test.

Variations	SS	df	MS	F	*p*
Between groups	35.34	1	35.34	0.24653	0.6234
Within groups	4014.78	28	143.38		

Storm drains are also pointing sources for plastic debris, where MPs originate from road markings and car tires abrasion in urban areas^61,62^. Therefore, Horton et al.^57^ reported MPs originated from vehicles in River Thames (UK). Remarkably, as reported by the Norwegian Environment Agency, MPs in aquatic ecosystems are dominant significant from tire wear and tear particles (53%), compared to other MPs in oceans^63^. Identifying and distinguishing various MPs contamination inputs are quite challenging due to heterogeneity in riverine ecosystems and are yet to be investigated. More comprehensive investigations of specific sources enable better understanding and their implications to catchment-level plastic emission and discharges, allowing to implement suitable mitigation strategies.

Statistics of MPs abundance in Bangladesh are yet to be investigated. To date, very few investigations have been conducted on the beaches of Bangladesh and the Bay of Bengal. Those studies have publicized the sediments of these coastal areas are being polluted with huge MPs. No such MPs research was performed yet in the river estuary of Bangladesh, which is situated near the Bay of Bengal. MPs pollution in different estuaries worldwide was summarized in Table 3 and observed MPs abundance from few to thousands of particles per kilogram.

**Table 3:** Summary for MPs contamination in various estuarine sediments reported worldwide.

Location	Country	Sample type	MP range/average	Polymer types	References
Estuary, UK	UK	Sediment	31 particles kg^−1^	Acrylic, alkyd, PE, PP, PA (nylon), PEST, polymethylacrylate, PP, PVA	Thompson et al.^64^
Five urban estuaries of KwaZulu-Natal	South Africa	Sediment	745.4 ± 129.7 particles	Not identified	Naidoo et al.^65^
Pearl River Estuary	Hong Kong	Sediment	5595 ± 27,417 items	Fragments, pellets, and expanded polystyrene (EPS)	Fok and Cheung^66^
Gulf of Mexico estuary		Sediment	5–117 items m^−2^	PP, PE, PS, PEST, aliphatic PA	Wessel et al.^67^
Solent estuarine complex	UK	Sediment	2759 particles	PE, PP, PVC	Gallagher et al.^68^
Vembanad Lake	India	Sediment	252.8 particles m^−2^	PP, PS, HDPE, LDPE	Sruthy et al.^69^
Jagir Estuary,	Surabaya City, Indonesia	Sediment	590 particles·kg^−1^	LDPE, PP, PES	Firdaus, et al.^70^
Brisbane River	Australia	Sediment	10–520 items kg^−1^	PE, PA, PP, PET	He et al.^71^
Awano River Ayaragi River Asa River Majime River	Japan	Surface water	132.80 ± 15.3 items L^−1^ 111.88 ± 21.42 items L^−1^ 130 ± 27.84 items L^−1^ 272.50 ± 299.15 items L^−1^	PP, PE, Vinylon PE, PET, Vinylon PP, PE, Vinylon PP, PS, PET	Kabir et al.^44^
Karnaphuli River Estuary	Bangladesh	Sediment	22.29–59.5 items kg^−1^	PE, PS Cellulose, PA (nylon), PET	The present study

*PE* polyethylene, *PA* polyamide, *PP* polypropylene, *PET* polyethylene terephthalate, *PS* polystyrene, *PEST* polyester, *PVC* polyvinyl-alcohol.

### Shape, color, size, and polymer types of microplastics

#### Shapes of MPs

**Figure 4:**
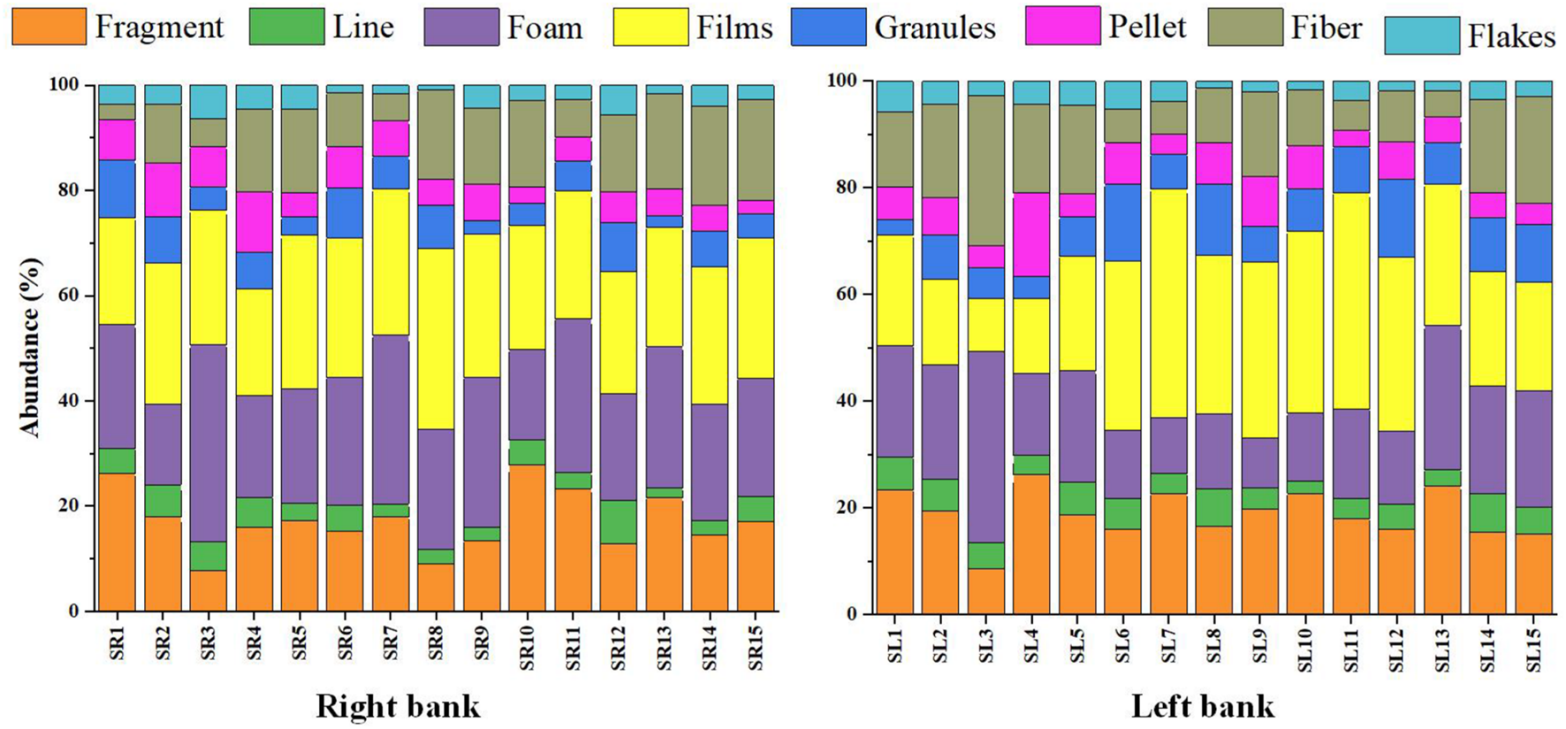
Proportions of MPs shape among sampling sites of right and left bank of Karnaphuli River estuary.

**Figure 5:**
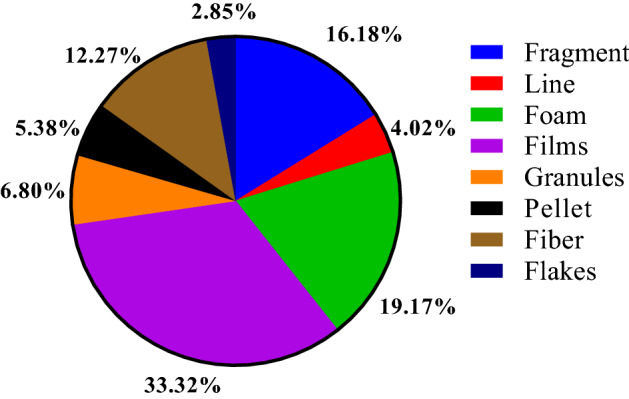
The overall percentage of MPs shapes found in the Karnaphuli River Estuary.

**Figure 6:**
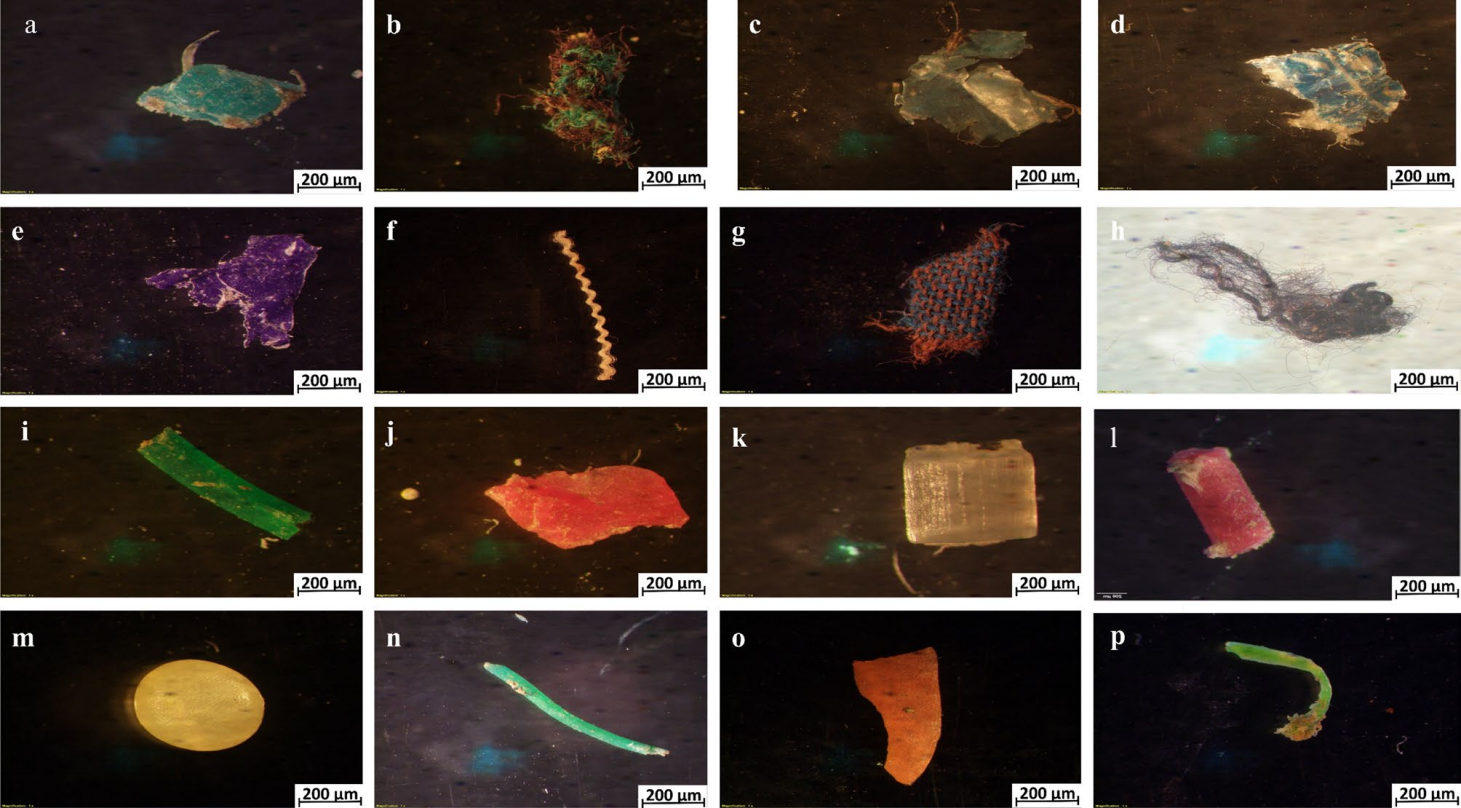
Representative images of different shapes and colors of MPs particles in sediment samples, films: (**a**, **d** and **e**), fragment: (**i**, **j**, **l**), fiber: (**b**, **f**–**h**), flakes: (**c**), line: (**n**, **p**), pellet: (**k**, **m**), foam: (**o**).

Most MPs found in the Karnaphuli River estuary are irregularly shaped and are sorted into fragments, fiber, foam, pellet, granules, lines, films, and flakes, on both right and left banks (Fig. 4). Films were the most dominant shape (33.32%) in the study area, followed by fragment (16.18%), foam (19.17%), fiber (12.27%), granules (6.80%), pellet (5.38%), line (4.02%) and flakes (2.85). MPs distribution varied from one site to another (Fig. 5). Sediment samples from the upper Estuary (Sites 13) revealed higher fiber compositions (65%) than the other samples in the river. Shapes of the MP particles are shown in Fig. 6.

#### Sizes

**Figure 7:**
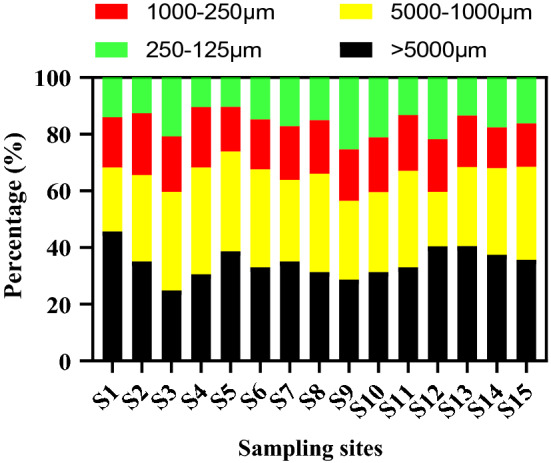
Proportions of MPs size range observed in sampling sites of Karnaphuli River Estuary.

MP sizes are crucial property that significantly depends upon the density and shapes since MPs are potentially ingested by numerous marine organisms^72–74^. Ingested MPs are unintentionally transferred to food chains^75,76^. Figure 7 depicts the station-wise distribution of MPs among different size categories. The observed MPs were categorized into size ranges of > 5000 µm, 5000–1000 µm, 1000–250 µm, and 250–125 µm. Most of the MPs measured were > 5000 µm (34.88%), followed by 5000–1000 µm (30.38%), 1000– µm (17.99%), and 250–125 µm (16.74%) (Fig. 7). Similar results were observed in previous findings published by^77–79^.

#### Polymer types

**Figure 8:**
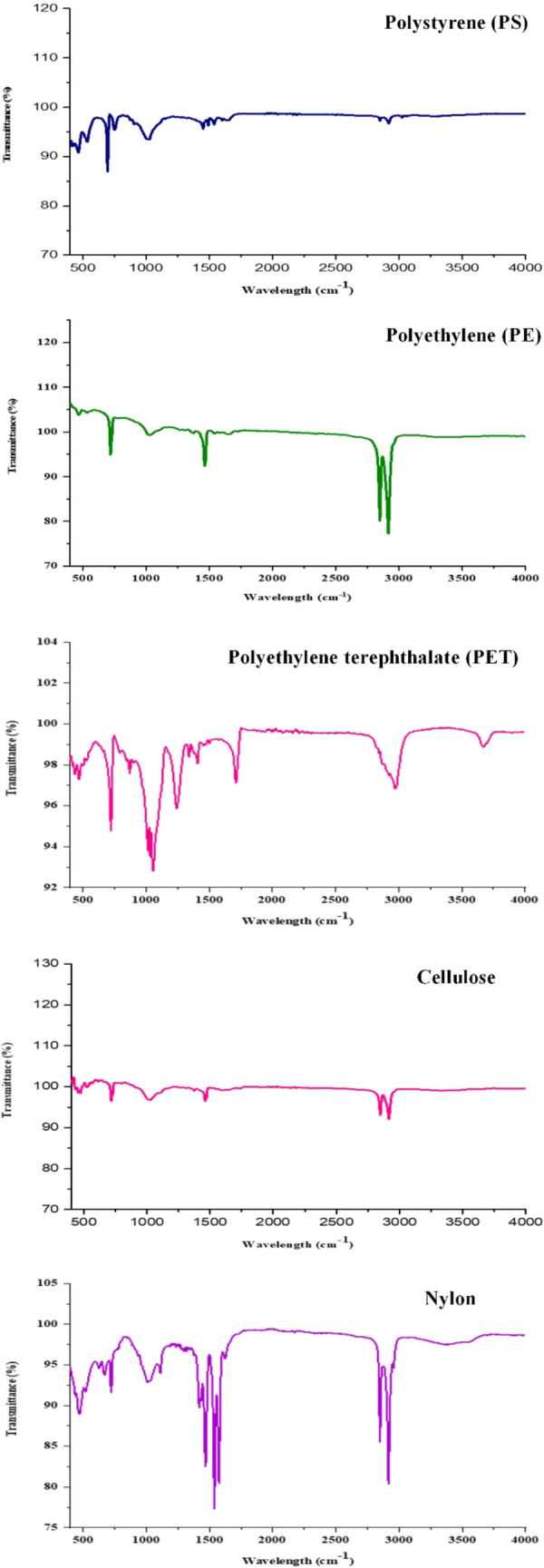
μ-Fourier transform infrared (FTIR) spectra of MPs observed in the river estuary.

**Figure 9:**
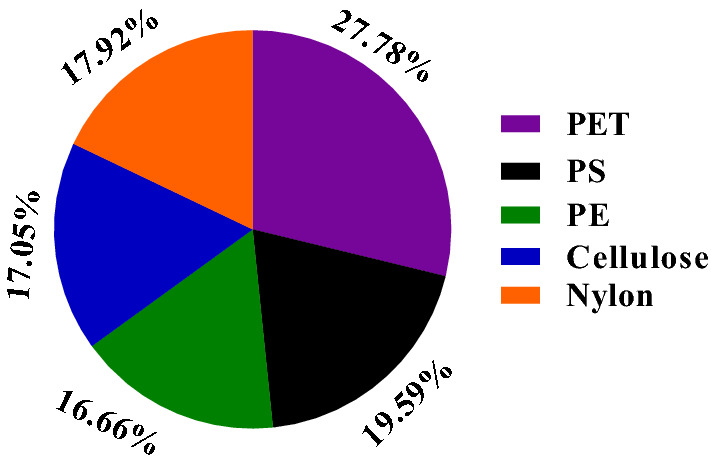
Percentage composition of MPs by polymer.

Polymeric characteristics of MPs were observed using FTIR analysis, which identified various polymers, such as cellulose, polyethylene (PE), polyethylene terephthalate (PET), polystyrene (PS), and nylon (Fig. 8). Based on FTIR analysis, PS, PE, PET, cellulose, and nylon accounted for 19.59, 16.66, 27.78, 17.05, and 17.92%, respectively (Fig. 9). PET was the maximum abundant polymer compared to PS, PE, cellulose, and nylon due to biofouling. Biofouling occurs due to increased MPs density and their weight, settling MP particles to sea beds^8,80–82^.

#### Color

**Figure 10:**
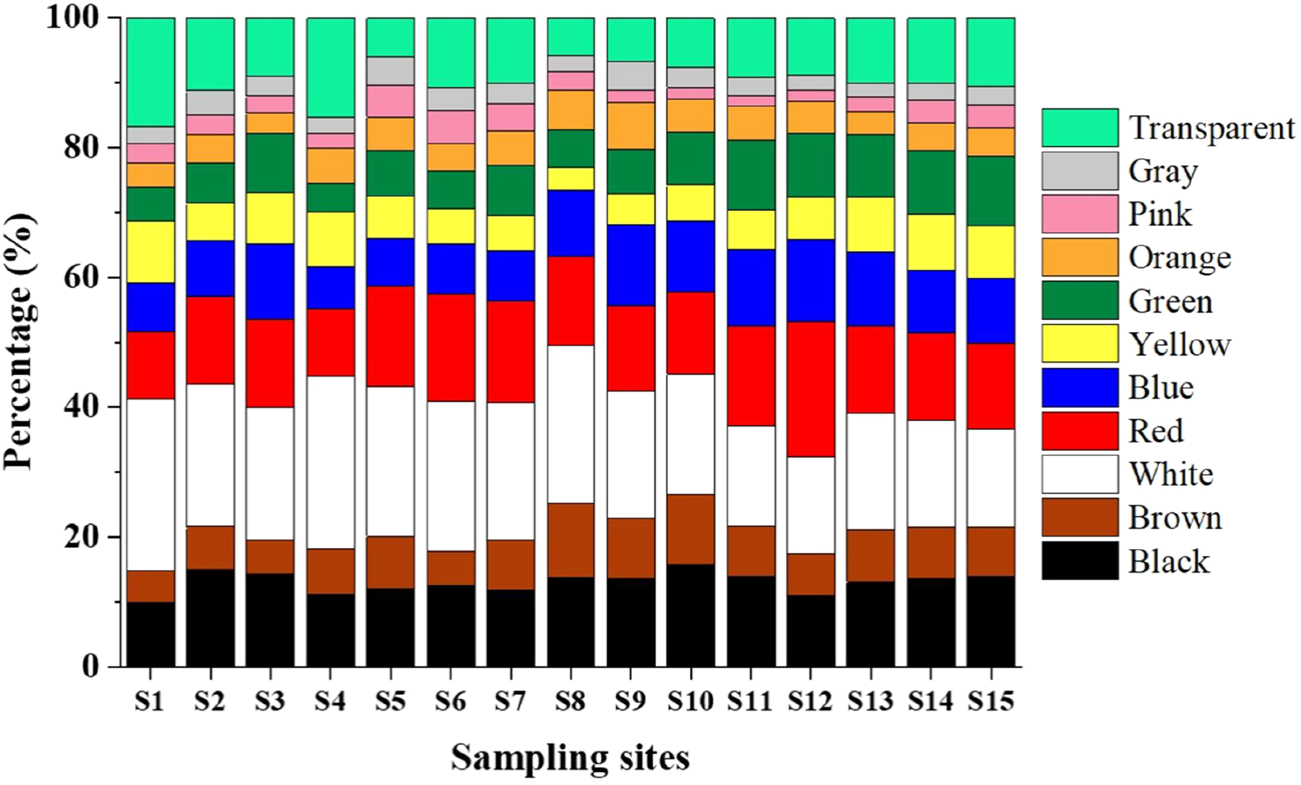
Proportions of MPs by colors in sampling sites of the river estuary.

MPs appearance makes unintentional ingestion by biota due to their possibly ingested, due to their resemblance with original prey or foodstuffs^52,83^. In Karnaphuli River Estuary, MPs of various colors are observed, like red, white, black, green, brown, yellow, orange, pink, gray, blue, and transparent (Fig. 10). Few studies have reported aquatic organisms to resemble foodstuffs with attractive MPs and make them available for ingestion due to their small particle sizes and high buoyancy in aquatic ecosystems^68,84^. White MPs were found maximum (19.25%) in the river estuary, followed by red, black, blue, transparent, green, etc., as shown in Fig. 10. It is observed that MPs with various colors sourced from plastics used in daily life, for example, clothes, packaging, fishing nets, etc., settled primarily on sediments^85,86^. On the other hand, MPs color also alters due to weathering during transport in aquatic ecosystems, surface water, and marine materials^87,88^. Therefore, colored MPs in the Karnaphuli River estuary recommend that these MPs originate from synthetic and organic materials; after that, in-depth investigations for detailed sources are required in the Karnaphuli River estuary.

### Source identifications

**Figure 11:**
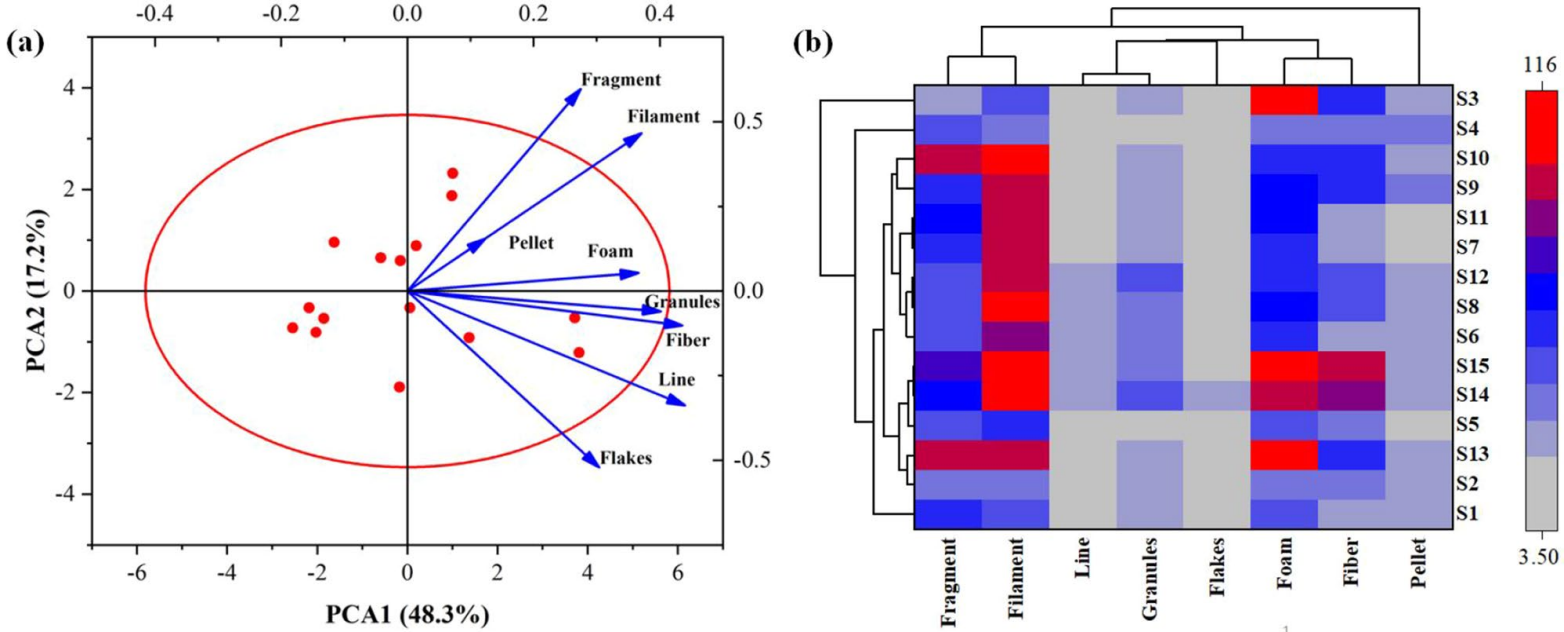
(**a**) Principal component analysis (PCA) based on shapes of MPs, and (**b**) clustering of various shapes with respect to sampling sites via heat map in the Karnaphuli River estuary. This heat map was constructed using Origin Pro.

Because of the proportions of various plastic types at different sample sites, using principal component analysis (PCA) to analyze the prevalence of MPs can be more understandable. PCAs of eight different MPs describe their distributions in the sediment of Karnaphuli River Estuary shown in Fig. 11a, which revealed the substantial differences via their distributions from each sampling site^89^. Results revealed that different MPs shapes dominate different sampling sites in the Karnaphuli River estuary. The first principal component analysis (PCA1) has shown robust contributions (48.3%) for fragments, films, pellet, and foam observed widely in sediments of many sampling sites in the Karnaphuli River estuary. Whereas second component (PCA2) showed granules, fiber, line, and flakes are dominated (17.2%) in sediments but possesses less distribution compared to PCA1, in which most MPs are originated from activities, like household, fishing, and agricultural practices, along with chemical and clothes industries. Thus, distribution characteristics have shown agricultural activities as the primary source for films in the river estuary. The eight parameters (shapes) were distinctly categorized into two groups, and heat map analysis was used to distinguish sampling sites with identical shape distributions (Fig. 11b). Results from the Karnaphuli River estuary indicated that foam, films, fragments, and pellet, as primary MPs sources in the Karnaphuli River Estuary, which was in accordance with various other research performed on freshwater ecosystems^90,91^. Moreover, distribution characteristics of different shapes of MPs are closely associated with agricultural, fishing, wastewater treatments, etc., in regional characteristics.

### Pollution risk assessment

**Figure 12:**
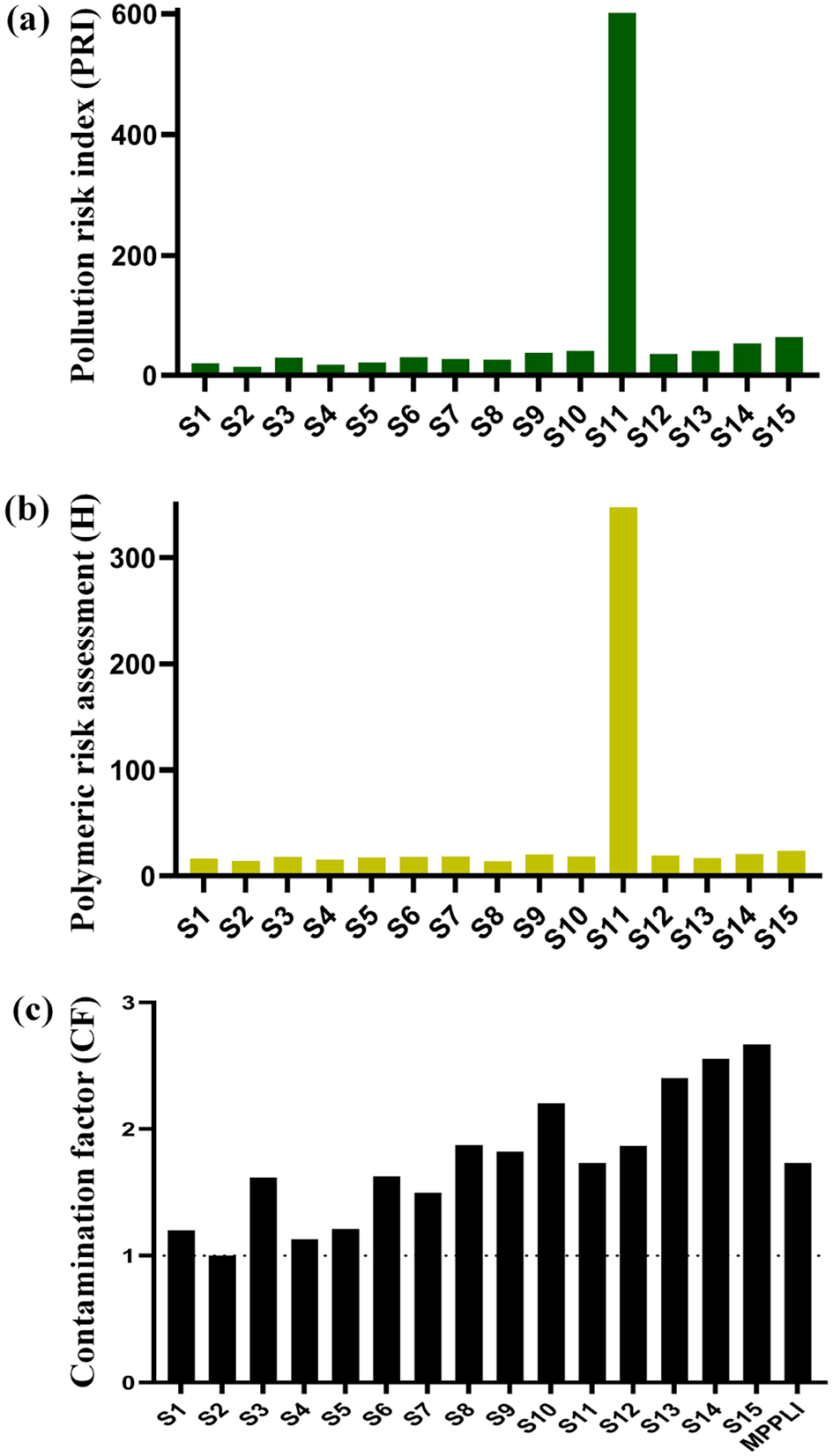
Microplastics risk (**a**) pollution risk index (PRI), (**b**) polymeric risk assessment (H), and (**c**) contamination factor (CF).

PRI values revealed low to very high risks among all sampling sites in the Karnaphuli River estuary (Fig. 12a). Midstream and downstream sampling sites, except for S11, were under low-risk zone (as PRI < 150). MPs from S11 site with PRI of 601.53 belong to the highly hazardous category. Considering land-uses, it was observed that the agricultural land-use dominating S11 site posed a highly hazardous category under the pollution risk index. Other sampling stations shown under low risk categories, e.g., sampling sites S1(19.72), S2 (14.06), S3 (28.79), S4 (17.26), S5 (21.19), S6 (29.29), S7 (27.11), S8 (25.99), S9 (36.96), S10 (40.40), S12 (37.72), S13 (40.39), S14 (52.57) and S15 (63.29) have shown PRI < 150. Besides, in downstream sampling sites, S12-S15 spread with industries, agricultural, residential, and urban land uses. For the entire study area, the PRI was 43.55, which indicates river estuary overall under the low-risk category. Therefore, in this study, as polymers are source-specific, as a result, land-use behavior affects the sources of polymers and results in high MPs pollution posed by point and non-point sources of MPs from various land-uses based on polymeric types as well as their toxicity.

Notably, polymeric risk assessments provided insights into the richness of toxic polymers in river environments. MPs polymer risk index (H) for all sampling sites are presented in Fig. 12b. The results showed that polymeric risks varied low to high from upstream to downstream. Based on the risks classification, only samples from S11 site showed considerable risks with polymeric risk assessment of 347, which indicated high-risk category (Class III) and revealed the presence of toxic polymers that results in higher hazard scores (Nylon 6: 50; PS: 30), along with the stations^47^. Even though highly toxic polymers were observed but in lower proportions, compared to polymers that possess higher polymeric hazard scores. Higher proportions of polymers with relatively low hazard scores resulted in lower polymeric risk values, for example, PS: 11; PET:4. However, polymeric risk assessment (H) for sampling site S2 showed low polymeric risks (14.06) of Class II from the entire river estuary.

MPs CF results clearly showed that all sampling sites in the river were significantly polluted with MPs (1 ≤ CF < 3) and indicated similar pollution levels at all sampling stations and are moderately contaminated with high MPs abundance (Fig. 12c). The overall pollution load index is 1.73 for the river estuary, which shows PLI > 1, indicating MPs pollution for all river sediment samples analyzed in the Karnaphuli River estuary. However, by stations, CF followed the decreasing order of contamination as S15 > S14 > S13 > S10 > S8 > S12 > S9 > S11 > S6 > S3 > S7 > S5 > S1 > S4 > S2. Contamination of sediment by microplastics is undesirable as it can accumulate in the fish and other organisms, causing deleterious effects.

## Conclusions

Estuaries are crucial ecosystems to understand the fate and transport of MPs to the ocean via land-based sources. This was the first study on MPs pollution from Karnaphully River estuarine ecosystem in Bangladesh. In this study, a high abundance of MPs was observed in the Karnaphuli estuary compared to other most of the estuaries in the Asian region and found gradually higher levels towards the mouth of the estuary. Film-shaped, white-colored, and larger-sized (1000–5000 μm) MPs were dominant in the Karnaphully River estuary. Among the polymer types, polyethylene terephthalate was most abundant. The pollution load and risk indices showed that all sampling sites across river banks were polluted with MPs and posed significant risks to the ecosystems. Land-use behavior such as agricultural runoff, industrial effluents, household & fishing activities, and urbanized locality dominate polymeric abundance. This study provides a database baseline for microplastic pollution in the Karnaphully River estuary due to anthropogenic activities. Thus, policymakers, scientists, ecologists, social environmentalists, and hydrologists are based on the database non-government organizations, government, etc., can strategically plan for river estuary conservation and management.

## Data Availability

The datasets used and/or analyzed during the current study are available from the corresponding author on reasonable request.
